# The association of umbilical cord blood neurofilament light with non‐reassuring fetal status: A prospective observational study

**DOI:** 10.1002/ijgo.70421

**Published:** 2025-08-27

**Authors:** David Zalcberg, Kaitlin Kramer, Emma Payne, Thomas Payne, Shreeya Marathe, Neha Mahajan, Ashly Liu, Jessica Barry, Andrew Duckworth, Mitchell Brooks, Bradley de Vries, Fernando Gonzalez‐Ortiz, Kaj Blennow, Henrik Zetterberg, Adrienne Gordon, Benjamin Moran, Helen J. Manning, Robert D. Sanders

**Affiliations:** ^1^ Central Clinical School, Faculty of Medicine and Health The University of Sydney Camperdown New South Wales Australia; ^2^ Department of Anesthetics, Royal Prince Alfred Hospital Sydney Local Health District Camperdown New South Wales Australia; ^3^ St George Hospital South Eastern Sydney Local Health District Caringbah New South Wales Australia; ^4^ The Royal Melbourne Hospital Parkville Victoria Australia; ^5^ Sydney Institute for Women, Children and their Babies Sydney Local Health District Camperdown New South Wales Australia; ^6^ NHMRC Clinical Trials Center The University of Sydney Camperdown New South Wales Australia; ^7^ Department of Psychiatry and Neurochemistry, Institute of Neuroscience and Physiology The Sahlgrenska Academy at the University of Gothenburg Mölndal Sweden; ^8^ Clinical Neurochemistry Laboratory Sahlgrenska University Hospital Mölndal Sweden; ^9^ Paris Brain Institute, ICM, Pitié‐Salpêtrière Hospital Sorbonne University Paris France; ^10^ Neurodegenerative Disorder Research Center, Division of Life Sciences and Medicine, and Department of Neurology, Institute on Aging and Brain Disorders University of Science and Technology of China and First Affiliated Hospital of USTC Hefei People's Republic of China; ^11^ Department of Neurodegenerative Disease UCL Institute of Neurology London UK; ^12^ UK Dementia Research Institute at UCL London UK; ^13^ Hong Kong Center for Neurodegenerative Diseases Clear Water Bay Hong Kong China; ^14^ Wisconsin Alzheimer's Disease Research Center, University of Wisconsin School of Medicine and Public Health University of Wisconsin‐Madison Madison Wisconsin USA; ^15^ Critical Care Program The George Institute of Global Health Sydney New South Wales Australia; ^16^ Department of Intensive Care Gosford Hospital Gosford New South Wales Australia; ^17^ Department of Anesthesia and Pain Medicine Gosford Hospital Gosford New South Wales Australia; ^18^ School of Medicine and Public Health University of Newcastle Callaghan New South Wales Australia; ^19^ Department of Obstetrics and Gynecology Central Coast Local Health District Gosford New South Wales Australia; ^20^ Institute of Academic Surgery, Royal Prince Alfred Hospital Sydney Local Health District Camperdown New South Wales Australia

**Keywords:** asphyxia, HIE, hypoxic–ischemic encephalopathy, neurofilament light, umbilical cord blood

## Abstract

**Objective:**

Early detection of hypoxic–ischemic encephalopathy (HIE) in neonates is critical. We conducted a pilot cohort study to determine the feasibility of collecting umbilical cord blood samples for neurofilament light (NfL) and to assess the association of NfL with non‐reassuring fetal status and other cord biomarkers. We aimed to address (1) Feasibility of cord NfL sample collection and analysis; (2) Association of NfL with non‐reassuring fetal status (CTG changes and/or documented non‐reassuring fetal status), neonatal intensive care unit (NICU) admission and length of stay; (3) Correlation of NfL with other cord biomarkers.

**Methods:**

This was a prospective cohort study performed in a single, large tertiary and quaternary referral hospital. A total of 108 maternal participants consented to donate cord blood. Umbilical cord blood NfL levels were measured via single molecule array (Simoa) analysis.

**Results:**

Cord NfL was higher in preterm neonates and was correlated with cord lactate, pH, and base excess. After controlling for mode of delivery and gestational age, NfL (odds ratio [OR] = 2.29, 95% confidence interval [CI]: 1.15–5.57), but not pH (OR = 0.78, 95% CI: 0.42–1.41), base excess (OR = 0.83, 95% CI: 0.37–1.86), or lactate (OR = 1.06, 95% CI: 0.51–2.12) was associated with non‐reassuring fetal status. NfL levels were higher in neonates admitted to NICU (median [IQR]: 11.3 [7] vs 8.5 [5.1]).

**Conclusion:**

Cord blood NfL analysis was feasible and provided correlates of adverse outcomes. Higher venous cord blood NfL levels were associated with non‐reassuring fetal status. Further research is needed to validate these findings and establish the role of NfL, if any, in clinical practice.

## 1 INTRODUCTION

Hypoxic–ischemic encephalopathy (HIE) is a common cause of neonatal encephalopathy (NE), occurring in 1–2 per 1000 live births.^1^ Therapeutic hypothermia is the only proven treatment for HIE,^2^, ^3^, ^4^, ^5^ and must be started within 6 h of birth.^1^ Current practice for identifying HIE relies on clinical signs, including need for resuscitation and cord blood acid–base analysis, which has limited sensitivity in part because cord blood acid–base biomarkers are non‐specific for detection of neuronal injury.^6^, ^7^ Identification of in utero fetal hypoxia can help to direct expedited delivery of the fetus, in an attempt to decrease the incidence of HIE. A composite outcome “non‐reassuring fetal status” may be used as an indicator of fetal hypoxia, and is defined by abnormalities in fetal heart rate (primarily detected by cardiotocography [CTG]), fetal scalp blood sampling, and ultrasound parameters.^8^, ^9^

Neurofilament light (NfL) is a neuronal protein released at the time of many types of neuronal injuries.^10^ Two case–control studies of cord NfL have suggested associations with HIE and perinatal morbidity.^11^, ^12^ However, research is lacking regarding the correlation between cord NfL and non‐reassuring fetal status.^13^ Demonstration of such an association would reinforce the use of NfL as a possible adjunct in the diagnosis of HIE, and support continued development of a rapid point‐of‐care scalp NfL test that may, in future, help identify in utero hypoxia.

We conducted a prospective cohort study to determine the association of umbilical cord NfL with various perinatal factors. Our hypotheses were: (1) Collection and analysis of cord blood NfL is feasible; (2) Cord NfL has a stronger association with non‐reassuring fetal status than other cord biomarkers; and (3) Cord NfL is correlated with other cord markers of non‐reassuring fetal status.

## 2 MATERIALS AND METHODS

### 2.1 Study design

A prospective cohort study was conducted with women who birthed at Royal Prince Alfred Hospital (RPAH). RPAH is an inner‐city tertiary and quaternary public referral hospital in Sydney with mostly urban‐dwelling patients. Participants were prospectively recruited at any time from 4 weeks prior to estimated delivery date, to date of delivery (including during delivery). Informed consent was obtained from the birth parent for all participants. Study data were collected and stored in REDCap by the research team. Ethics approval was obtained from Sydney Local Health District Human Research and Ethics Committee (approval no. 2022/ETH01100). Data are reported in accordance with the Strengthening the Reporting of Observational Studies in Epidemiology (STROBE) guidelines.^14^

### 2.2 Study population

Women who birthed at RPAH from October 2022 were eligible for study recruitment, including all live births delivered vaginally or via cesarean section (CS). Women were excluded if they were non‐English speaking or had a history of psychological illness, or other conditions that interfered with capacity to provide informed consent.

### 2.3 Biospecimen collection and analysis

Immediately at birth, 3 mL of umbilical venous cord blood was collected in EDTA tubes from each study participant. Blood samples were centrifuged in the Department of Anesthetics laboratory, and plasma aliquots stored in barcoded cryovials.

Plasma samples were sent to the University of Gothenburg in Sweden for analysis. Plasma NfL concentration was measured using the NF‐Light assay on a single molecule array (Simoa) HD‐X instrument according to the manufacturer's instructions (Quanterix, Billerica, Massachusetts, USA). All measurements were done with a four‐fold dilution factor in singlicates and performed on one occasion using one batch of reagents with the analyst blinded to clinical data. Intra‐assay coefficients of variation were <10% derived from internal control plasma samples measured in duplicate on each analytical run.

### 2.4 Demographic and outcome data collection

Demographic and outcome data were extracted from electronic medical records. Data extraction included antenatal history, maternal and child health factors, intrapartum events and monitoring, adverse perinatal events, and any health events up to 4 weeks postpartum. Researchers collecting these data were blinded to the NfL results.

### 2.5 Outcomes

#### 2.5.1 Primary trial feasibility outcomes

Our primary feasibility outcomes were the proportion of eligible participants recruited to the study and the proportion of enrolled participants who had cord blood samples successfully sent for analysis.

#### 2.5.2 Primary clinical outcome

Our primary clinical outcome was the association of cord NfL with non‐reassuring fetal status. “Non‐reassuring fetal status” was defined as any record of non‐reassuring fetal status in the intrapartum or antenatal record of care, as well as any documented CTG changes aligning with “red zone” criteria per the NSW Health electronic fetal monitoring guidelines (Appendix S1). We adjusted our primary outcome for mode of delivery (vaginal or elective CS or emergency CS) and gestational age, given previous work has suggested that vaginal delivery and longer gestation are associated with greater cord NfL, and we hypothesized that these would also be associated with non‐reassuring fetal status.^13^

#### 2.5.3 Secondary clinical outcomes

Secondary outcomes included the association of NfL with other cord biomarkers (lactate, base excess, pH), intrapartum factors (mode of delivery, duration of labor), fetal factors (gestational age, head circumference, birth weight), and other neonatal outcomes (resuscitation requirement, neonatal intensive care unit [NICU] admission, NICU length of stay). A full list is provided in Appendix S2. We a priori defined preterm as birth at <37 weeks' gestation and very preterm as <32 weeks' gestation. We defined resuscitation requirement as any continuous positive airway pressure requirement postnatally. Analyses of birth weight and head circumference were adjusted for gestational age, as this has been suggested to be a confounder.^13^

### 2.6 Power analysis

Previous work has suggested the standard deviation (SD) of umbilical cord NfL is 13 pg/mL.^11^ To ensure that the 95% confidence interval (CI) estimate for mean NfL in our sample was within 3 pg/mL of the true population mean, 73 neonates were required. Anticipating possible issues with sample storage/shipment, 110 mothers were recruited. Using the previously reported incidence of non‐reassuring fetal status of approximately 16%,^8^ 110 participants yields 80% power to detect a large effect size (Cohen's *d* = 0.8), based on a two‐sided *t*‐test with *α* = 0.025 (halved from 0.05 to account for our dual feasibility/clinical primary outcome).

### 2.7 Statistical analysis

NfL concentrations showed a strong positive skew, so were log_10_ transformed for all analyses. Where the dependent variable was binary, we used logistic regression. Biomarker concentrations were standardized in these models by subtracting the mean and dividing by the standard deviation. Area under the receiver operator curve (AUROC) was calculated for biomarkers in predicting non‐reassuring fetal status. The 95% CI for the AUROC was calculated using 2000 stratified bootstrap replicates. The calculation of Youden's Index from the receiver–operator curve (ROC)^15^ was used to determine a binary cutoff value for all biomarkers that maximized the sum of the specificity and sensitivity. We did not undertake model calibration nor perform cross‐validation—our analyses aimed to be descriptive.

Regarding secondary outcomes, we report rank‐based nonparametric methods for bivariate biomarker analysis. Linear regression with ordinary least squares estimation was used for multivariable models where pH or base excess was the dependent variable. Where log_10_ NfL or lactate was the dependent variable, we used a generalized linear model with an inverse Gaussian family with log link. We aimed to model count data using the simplest possible distribution (the Poisson distribution), unless overdispersion was present, in which case we used a negative binomial family model. Nested models were compared using *F*‐tests (or Chi‐squared tests for count models). As this study was hypothesis‐generating, no adjustments were made for multiple comparisons.

All analyses were conducted in R using RStudio (version 2023.06.1; R Foundation for Statistical Computing, Vienna, Austria), using the lme4, plotROC, and pROC packages.

## 3 RESULTS

Cohort demographic information is summarized in Table S1.

### 3.1 Primary outcomes

#### 3.1.1 Feasibility of testing cord NfL

Of 509 births at RPAH over the period October 24, 2022 to December 9, 2022, 110 women were recruited, meaning 22% of all eligible participants were enrolled in the study. A total of 108 participants had cord blood sent for analysis (STROBE diagram: Figure S1), representing a 98% conversion rate from enrolment to analysis.

#### 3.1.2 Association of neurofilament light with non‐reassuring fetal status

Participants classified as experiencing non‐reassuring fetal status (*n* = 16) had higher cord NfL (median [Q1, Q3] NfL: 14.8 [11.2, 18.0] vs 8.4 [6.7, 11.4]). These participants also experienced higher lactate levels, as well as lower cord pH and base excess (Figure S2). The association between log_10_ NfL (in standardized form) and non‐reassuring fetal status persisted after adjusting for mode of delivery and gestational age (adjusted OR per 1 SD increase in log_10_ NfL = 2.29, 95% CI: 1.15–5.57; *P* = 0.038), whereas pH, base excess, and lactate were not associated with non‐reassuring fetal status in the adjusted model (Table 1).

**TABLE 1:** Results from binomial‐family models predicting non‐reassuring fetal status. Results for all four biomarkers are shown.

Characteristic	Log10 cord NfL^a^			Cord pH^b^			Cord base excess^c^			Cord lactate^d^		
	OR	95% CI	*P* value	OR	95% CI	*P* value	OR	95% CI	*P* value	OR	95% CI	*P* value
Unadjusted model												
Cord biomarker (standardized)	2.20	1.35, 4.00	0.004**	0.49	0.27, 0.85	0.013*	0.40	0.21, 0.70	0.002**	2.19	1.33, 3.74	0.003**
Model adjusted for mode of delivery and gestational age												
Cord biomarker (standardized)	2.29	1.15, 5.57	0.038*	0.78	0.42, 1.41	0.423	0.83	0.37, 1.86	0.650	1.06	0.51, 2.12	0.862

*Note*: Biomarkers were standardized by subtracting the mean and dividing by the standard deviation.

Abbreviations: AIC, Akaike information criterion; BIC, Bayesian information criterion; CI, confidence Interval; NfL, neurofilament light; OR, odds ratio.

^a^ Unadjusted model: AIC = 79.8; BIC = 85.1; No. Obs. = 105. Adjusted model: AIC = 70.1; BIC = 83.3; No. Obs. = 105; *P* value from *F*‐test comparing unadjusted vs adjusted model fit = 0.001.

^b^ Unadjusted model: AIC = 79.7; BIC = 85.0; No. Obs. = 104. Adjusted model: AIC = 70.2; BIC = 83.5; No. Obs. = 104; *P* value from *F*‐test comparing unadjusted vs adjusted model fit = 0.001.

^c^ Unadjusted model: AIC = 65.7; BIC = 70.8; No. Obs. = 94. Adjusted model: AIC = 62.1; BIC = 74.8; No. Obs. = 94; *P* value from *F*‐test comparing unadjusted vs adjusted model fit = 0.023.

^d^ Unadjusted model: AIC = 76.6; BIC = 81.9; No. Obs. = 103. Adjusted model: AIC = 70.8; BIC = 84.0; No. Obs. = 103; *P* value from *F*‐test comparing unadjusted vs adjusted model fit = 0.008.

* *P* < 0.05.

** *P* < 0.01.

**FIGURE 1:**
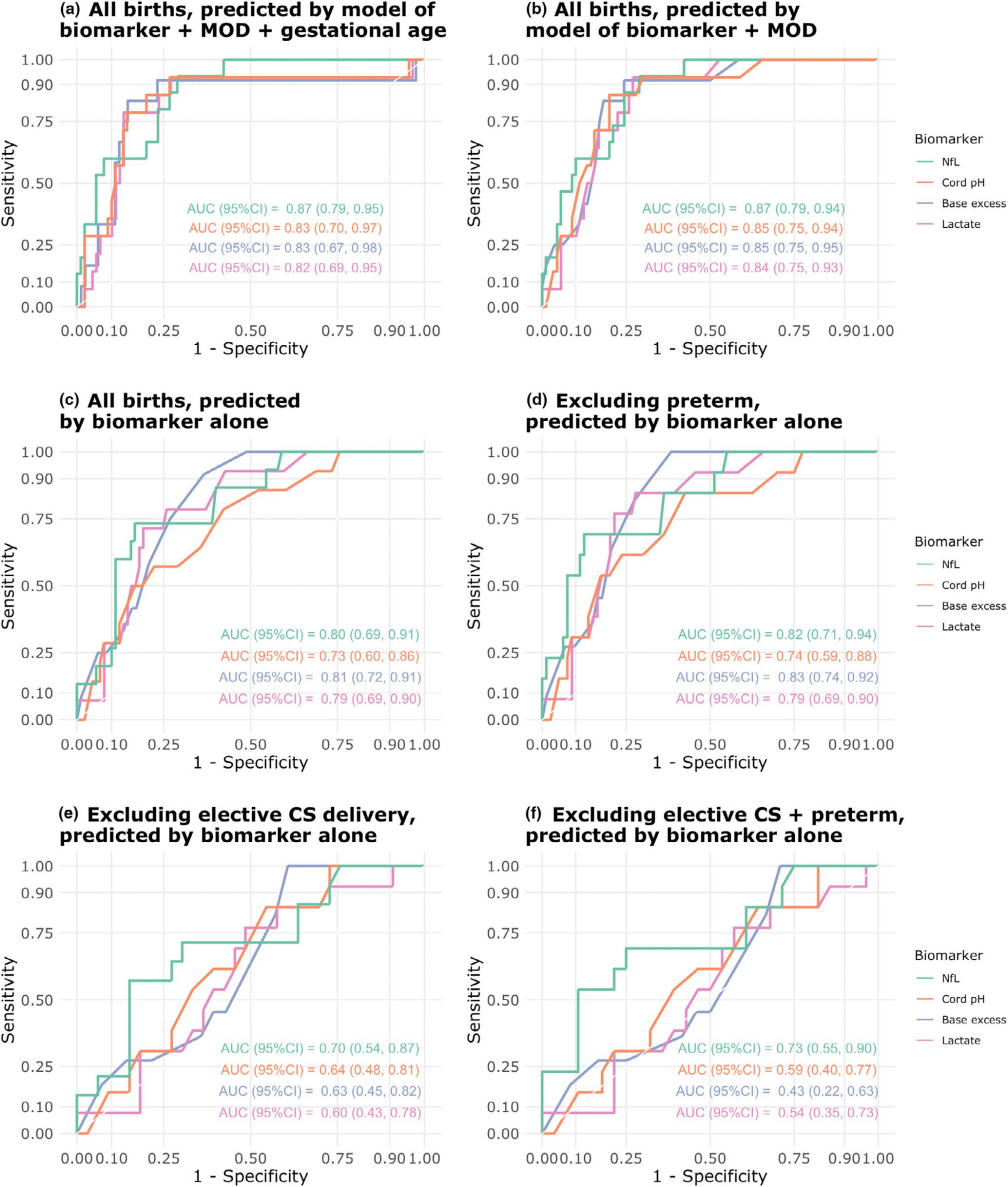
Receiver–operator curve (ROC) analysis of neurofilament light chain (NfL), pH, base excess, and lactate in predicting binary non‐reassuring fetal status. (a) ROC for the generalized linear model predicting non‐reassuring fetal status and including the regressors: biomarker, mode of delivery, and gestational age. (b) ROC for the generalized linear model predicting non‐reassuring fetal status and including the regressors: biomarker and mode of delivery. ROC for the biomarkers only, including (c) all births, (d) excluding preterm births, (e) excluding elective cesarean section (CS), and (f) excluding elective CS and preterm birth.

The combination of NfL with delivery mode (vaginal or elective CS or emergency CS) and gestational age provided a slightly better AUC (0.87 95% CI: 0.79–0.95) for the association with non‐reassuring fetal status than the combination of gestational age and delivery mode with the other biomarkers (Figure 1a). When using biomarkers alone for prediction of non‐reassuring fetal status, the most pronounced difference between NfL and other biomarkers was seen when excluding elective CS and preterm births (Figure 1f).

Youden's Index was calculated for all biomarkers. When including all births, diagnostic test characteristics of log_10_ NfL >1.09 included a negative predictive value of 0.96 (Table 2). In general, the specificity and positive predictive value of various log_10_ NfL cutoffs were much better than other biomarkers, while sensitivity was lower.

**TABLE 2:** Test characteristics of cord biomarkers in predicting non‐reassuring fetal status.

	Threshold (95% CI)^a^	Sensitivity (95% CI)	Specificity (95% CI)	Positive predictive value (95% CI)	Negative predictive value (95% CI)
All births					
Log10 NfL	1.09 (0.90, 1.17)	0.80 (0.60, 1.00)	0.84 (0.43, 0.92)	0.44 (0.23, 0.64)	0.96 (0.92, 1.00)
pH	7.25 (7.13, 7.31)	0.79 (0.43, 1.00)	0.66 (0.26, 0.91)	0.27 (0.17, 0.50)	0.95 (0.91, 1.00)
Base excess	−4.50 (−6.50, −3.50)	1.00 (0.75, 1.00)	0.63 (0.45, 0.83)	0.28 (0.21, 0.42)	1.00 (0.96, 1.00)
Lactate	3.95 (3.35, 5.00)	0.86 (0.64, 1.00)	0.75 (0.51, 0.88)	0.34 (0.23, 0.50)	0.97 (0.93, 1.00)
Excluding preterm births					
Log10 NfL	1.09 (0.90, 1.16)	0.77 (0.54, 1.00)	0.86 (0.44, 0.95)	0.48 (0.22, 0.72)	0.96 (0.93, 1.00)
pH	7.25 (7.17, 7.25)	0.77 (0.46, 1.00)	0.68 (0.45, 0.90)	0.29 (0.20, 0.50)	0.96 (0.91, 1.00)
Base excess	−4.50 (−6.50, −4.50)	1.00 (0.82, 1.00)	0.66 (0.52, 0.82)	0.30 (0.24, 0.44)	1.00 (0.97, 1.00)
Lactate	3.95 (3.35, 5.00)	0.85 (0.69, 1.00)	0.76 (0.52, 0.87)	0.37 (0.24, 0.52)	0.97 (0.93, 1.00)
Excluding elective CS births					
Log10 NfL	1.12 (0.90, 1.17)	0.71 (0.43, 1.00)	0.79 (0.27, 0.97)	0.58 (0.37, 0.87)	0.86 (0.77, 1.00)
pH	7.25 (7.12, 7.32)	0.92 (0.46, 1.00)	0.48 (0.18, 0.88)	0.40 (0.33, 0.63)	0.94 (0.79, 1.00)
Base excess	−4.50 (−11.50, −4.00)	1.00 (0.45, 1.00)	0.43 (0.25, 0.93)	0.41 (0.34, 0.75)	1.00 (0.81, 1.00)
Lactate	4.45 (3.35, 7.25)	0.85 (0.31, 1.00)	0.52 (0.21, 0.94)	0.41 (0.32, 0.71)	0.89 (0.76, 1.00)
Excluding elective CS and preterm births					
Log10 NfL	1.11 (0.90, 1.22)	0.69 (0.38, 1.00)	0.82 (0.32, 1.00)	0.65 (0.39, 1.00)	0.85 (0.76, 1.00)
pH	7.25 (7.08, 7.31)	0.85 (0.31, 1.00)	0.46 (0.11, 0.93)	0.42 (0.34, 0.69)	0.88 (0.73, 1.00)
Base excess	−9.50 (–Inf, Inf)	0.73 (0.00, 1.00)	0.46 (0.00, 1.00)	0.36 (0.31, 0.50)	0.76 (0.69, 1.00)
Lactate	4.45 (2.94, 9.55)	0.85 (0.08, 1.00)	0.50 (0.11, 1.00)	0.43 (0.33, 1.00)	0.83 (0.70, 1.00)

*Note*: The cutoff value is determined by the calculation of Youden's Index from the receiver–operator curve, which maximizes the sum of the sensitivity + specificity.

Abbreviations: CI, confidence interval; CS, cesarean section; NfL, neurofilament light.

^a^ Obtained using the calculation of Youden's Index (maximum sum of sensitivity + specificity).

### 3.2 Secondary outcomes

**FIGURE 2:**
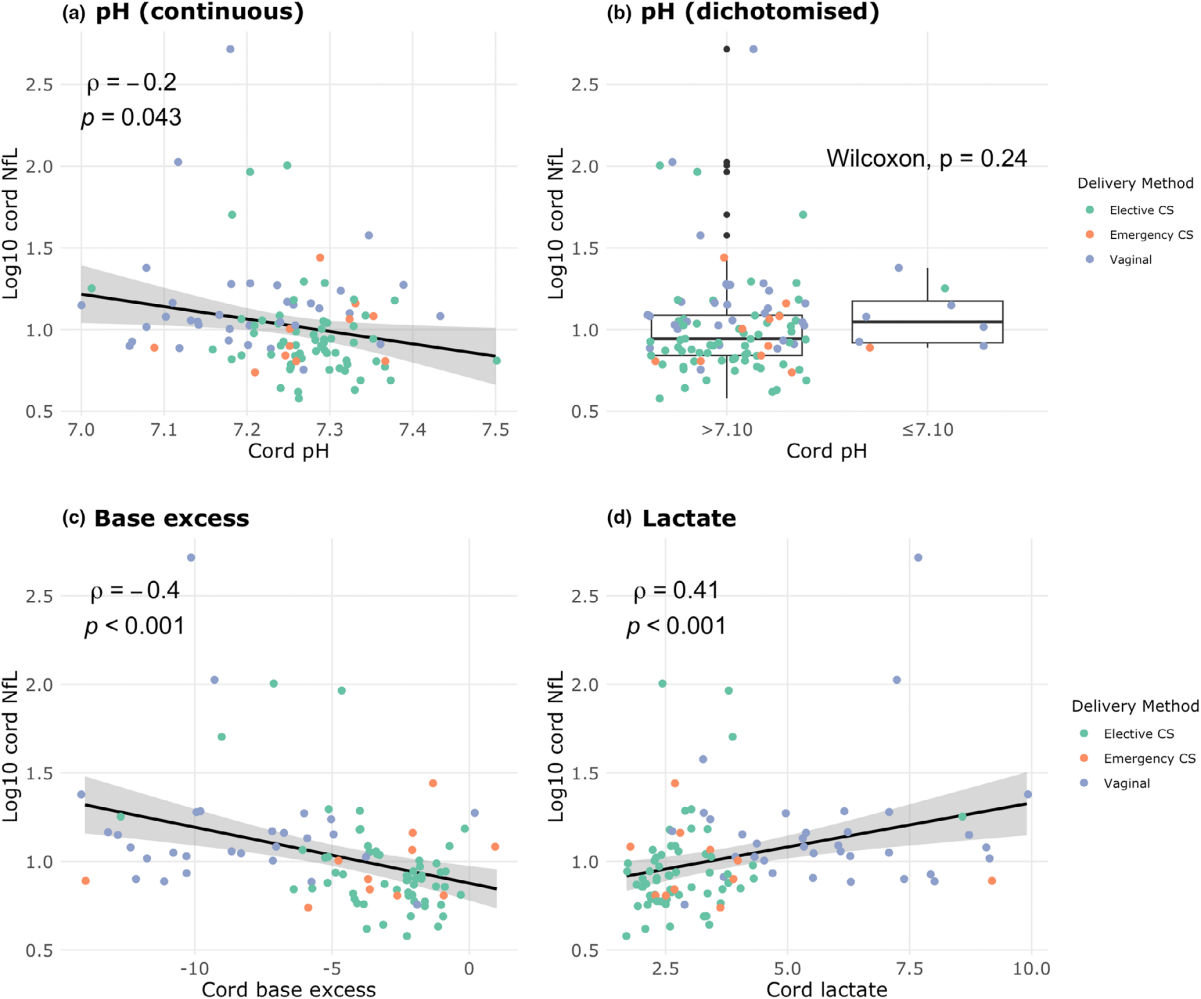
Correlation between cord blood biomarkers. Association of neurofilament light chain (NfL) (*n* = 108), (a) pH (*n* = 107), (b) pH ≤7.10 or pH >7.10 (*n* = 8; *n* = 99), (c) base excess (*n* = 97), and (d) lactate (*n* = 106). Spearman correlation coefficients are shown for continuous variables (a, c, d), Wilcoxon *P* values are shown for comparisons between individual modes of delivery (b). Green dot: elective cesarean section (CS); orange dot: emergency CS; purple dot: vaginal delivery.

Cord NfL demonstrated an inverse correlation with cord pH and base excess, and a positive correlation with cord blood lactate (Figure 2). Conversely, examining only participants who laboured, no association was found for any biomarker (Figure S3).

**FIGURE 3:**
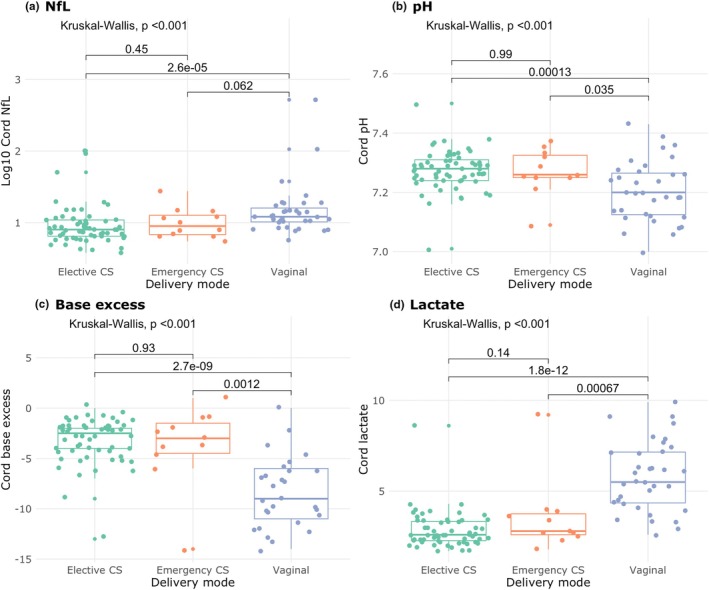
Comparison of neurofilament light chain (NfL) by mode of delivery—elective cesarean section (CS), emergency CS and vaginal delivery. Kruskal–Wallis *P* values shown. Green dot: elective CS; orange dot: emergency CS; purple dot: vaginal delivery.

NfL, pH, base excess, and lactate values differed significantly by mode of delivery (Kruskal–Wallis, all *P* < 0.001) (Figure 3). There was no statistically significant correlation of any biomarker with duration of first stage of labor. Whilst duration of the second stage was negatively correlated with pH (Spearman ρ = −0.49, *P* = 0.009) and base excess (Spearman ρ = −0.44, *P* = 0.044) and positively correlated with lactate (Spearman ρ = 0.40, *P* = 0.037), there was no statistically significant correlation with NfL (Spearman ρ = −0.27, *P* = 0.17) (Figure S4).

We observed a lower cord NfL level in participants who were born term (*n* = 94) compared to those born preterm (32−37 completed weeks gestation; *n* = 10), and very preterm (under 32 completed weeks' gestation; *n* = 4) (median [Q1, Q3] log_10_ NfL = 0.94 [0.84, 1.08] vs 1.18 [0.97, 1.48] vs 1.17 [1.14, 1.24]; Kruskal–Wallis *P* = 0.004) (Figure S5). Conversely, we did not observe a difference in cord pH, base excess, or lactate across these groups of gestational age.

Keeping gestational age constant, every 1 kg increase in birth weight was associated with an 11% decrease in mean log_10_ NfL (exp[*β*] = 0.89, 95% CI: 0.81–0.98; *P* = 0.025) (Table S2). There was no significant relationship with biomarkers and head circumference in adjusted analysis (Table S3).

We did not observe evidence of an association of NfL, pH, base excess, or lactate with resuscitation requirement after adjusting for preterm birth (Table S4). NfL values were significantly higher in participants who required NICU admission (median [Q1, Q3] log_10_ NfL: 1.05 [0.91, 1.18] vs 0.93 [0.84, 1.08]; Wilcoxon *P* = 0.040). Conversely, we did not observe evidence for a difference in cord pH and lactate values in neonates admitted to NICU versus those not, while base excess values were slightly higher in those admitted to NICU (Figure S6).

We did not observe strong evidence to suggest higher NfL values (in standardized form) were associated with an increased NICU length of stay in the unadjusted model (incidence rate ratio [IRR] = 3.04, 95% CI: 0.94–9.80; *P* = 0.063), or after adjusting for gestational age and mode of delivery (IRR = 1.51, 95% CI: 0.71–3.19; *P* = 0.284) (Table S5). Cord pH, base excess, and lactate were all associated with NICU length of stay in the unadjusted models, but none were statistically significant in the adjusted model (Table S5).

## 4 DISCUSSION

We observed an association between higher cord blood NfL with non‐reassuring fetal status, which remained robust after adjusting for mode of delivery and gestational age. In addition, we showed that NfL correlates with cord biomarkers of neonatal asphyxia. Together, our findings provide valuable evidence of the associations between NfL and surrogates of in utero hypoxia, which is supportive of future studies of NfL in the diagnosis and treatment of HIE.

The largest descriptive study of cord blood NfL to date (*n* = 665), by Kürner and colleagues, supports our findings of higher NfL levels in vaginal delivery.^13^ Meanwhile, in that study, higher gestational age was associated with greater cord NfL, while in our cohort, there was a negative association. In addition, our study found a negative association between NfL and birth weight, whilst Kürner and colleagues found no evidence of this association. A possible reason for these discrepancies is that Kürner and colleagues included only healthy term neonates,^13^ whilst we included a higher risk cohort (24% admitted to NICU), and 13% of our cohort were born preterm. NfL appears to be higher in preterm neonates, who also have lower birth weight.

Our study adds to previous research regarding the association between NfL and adverse outcomes in neonates. Studies of cord NfL in HIE are limited to case–control designs given the rarity of overt HIE.^11^, ^12^ The two case–control studies (*n* = 38 and 150) of cord blood demonstrated a higher NfL in cases of asphyxia with or without HIE,^11^, ^12^ with asphyxia defined as Apgar ≤7 at 5 min and/or umbilical cord blood acidosis (pH <7.0). By linking cord NfL with non‐reassuring fetal status, we attempt to bridge the results of these case–control studies to a possible role of NfL in the identification of in utero hypoxia, which may in future lead to studies of NfL in the diagnosis of milder or unrecognized cases of HIE. “Pathologic” fetal heart rate abnormalities, detected by CTG and ultrasound, may indicate early compensatory changes to hypoxia,^16^ and correlate with neonatal acidosis.^17^, ^18^, ^19^ Other studies of the plasma and CSF obtained in the days following birth have shown associations of NfL with neonatal asphyxia and HIE,^20^, ^21^ providing mechanistic support for our work.

Limitations of this study included a relatively small sample size (*n* = 108). In addition, our population included a high proportion of elective CS, which are excluded from intrapartum identification of non‐reassuring fetal status by our criteria. In addition, we did not assess whether hypoxia suggested by non‐reassuring fetal status led to downstream neurologic effects. There is not yet evidence that increased intrapartum monitoring and identification of non‐reassuring fetal status leads to decreased incidence of HIE overall, despite correlating with a reduction in neonatal seizure incidence.^22^, ^23^, ^24^, ^25^ Moreover, fetal heart rate patterns have differing clinical implications depending on fetal and maternal background, and therefore having variable sensitivity and specificity for hypoxic injury.

## 5 CONCLUSION

In conclusion, our study underscores the potential of NfL as a promising biomarker of non‐reassuring fetal status, and hence fetal hypoxia.

## AUTHOR CONTRIBUTIONS

DZ and RDS designed the study in consultation with BdV, KK, BM, and HM. FG‐O, HZ and KB supplied the assays and managed biofluid analysis. EP and TP conducted the statistical analysis. EP and DZ drafted the manuscript. All authors provided critical feedback on the manuscript.

## FUNDING INFORMATION

The work is supported by Sydney Local Health District and University of Sydney. KB is supported by the Swedish Research Council (#2017‐00915 and #2022‐00732), the Swedish Alzheimer Foundation (#AF‐930351, #AF‐939721, #AF‐968270, and #AF‐994551), Hjärnfonden, Sweden (#FO2017‐0243 and #ALZ2022‐0006), the Swedish state under the agreement between the Swedish government and the County Councils, the ALF‐agreement (#ALFGBG‐715986 and #ALFGBG‐965240), the European Union Joint Program for Neurodegenerative Disorders (JPND2019‐466‐236), the Alzheimer's Association 2021 Zenith Award (ZEN‐21‐848495), the Alzheimer's Association 2022‐2025 Grant (SG‐23‐1038904 QC), La Fondation Recherche Alzheimer (FRA), Paris, France, and the Kirsten and Freddy Johansen Foundation, Copenhagen, Denmark. HZ is a Wallenberg Scholar and a Distinguished Professor at the Swedish Research Council supported by grants from the Swedish Research Council (#2023‐00356; #2022‐01018 and #2019‐02397), the European Union's Horizon Europe research and innovation program under grant agreement no. 101053962, Swedish State Support for Clinical Research (#ALFGBG‐71320), the Alzheimer Drug Discovery Foundation (ADDF), USA (#201809‐2016862), the AD Strategic Fund and the Alzheimer's Association (#ADSF‐21‐831376‐C, #ADSF‐21‐831381‐C, #ADSF‐21‐831377‐C, and #ADSF‐24‐1284328‐C), the Bluefield Project, Cure Alzheimer's Fund, the Olav Thon Foundation, the Erling‐Persson Family Foundation, Stiftelsen för Gamla Tjänarinnor, Hjärnfonden, Sweden (#FO2022‐0270), the European Union's Horizon 2020 research and innovation program under the Marie Skłodowska‐Curie grant agreement no. 860197 (MIRIADE), the European Union Joint Program – Neurodegenerative Disease Research (JPND2021‐00694), the National Institute for Health and Care Research University College London Hospitals Biomedical Research Center, and the UK Dementia Research Institute at UCL (UKDRI‐1003).

## CONFLICT OF INTEREST STATEMENT

KB has served as a consultant and at advisory boards for Acumen, ALZPath, AriBio, BioArctic, Biogen, Eisai, Lilly, Moleac Pte. Ltd., Novartis, Ono Pharma, Prothena, Roche Diagnostics, and Siemens Healthineers; has served at data monitoring committees for Julius Clinical and Novartis; has given lectures, produced educational materials and participated in educational programs for AC Immune, Biogen, Celdara Medical, Eisai and Roche Diagnostics; and is a co‐founder of Brain Biomarker Solutions in Gothenburg AB (BBS), which is a part of the GU Ventures Incubator Program, outside the work presented in this paper. HZ has served at scientific advisory boards and/or as a consultant for Abbvie, Acumen, Alector, Alzinova, ALZPath, Amylyx, Annexon, Apellis, Artery Therapeutics, AZTherapies, Cognito Therapeutics, CogRx, Denali, Eisai, Merry Life, Nervgen, Novo Nordisk, Optoceutics, Passage Bio, Pinteon Therapeutics, Prothena, Red Abbey Labs, reMYND, Roche, Samumed, Siemens Healthineers, Triplet Therapeutics, and Wave, has given lectures in symposia sponsored by Alzecure, Biogen, Cellectricon, Fujirebio, Lilly, and Roche, and is a co‐founder of Brain Biomarker Solutions in Gothenburg AB (BBS), which is a part of the GU Ventures Incubator Program (outside submitted work). The other authors have no conflicts of interest to declare.

## Supporting information


Appendix S1.

Data S1.

## Data Availability

The raw data are available from the corresponding author upon reasonable request.
